# Supplementary material for: Effect of voluntary human mobility restrictions on vector-borne diseases during the COVID-19 pandemic in Japan: A descriptive epidemiological study using a national database (2016 to 2021)
Source: PLoS One. 2023 May 25;18(5):e0285107. doi: 10.1371/journal.pone.0285107 (PMC10212128; doi:10.1371/journal.pone.0285107)
Supplement: S1 Fig — a) Indices of travel agency activity. b) Indices of the passenger transportation industry activity. The declaration of a state of emergency was implemented from April 7 to May 25, 2020; from January 8 to March 21, 2021; from April 25 to June 20, 2021; and from July 12 to September 30, 2021. The quasi-emergency measure was implemented from April 5 to September 30, 2021, and from January 9 to March 1, 2022. (PPTX) [file pone.0285107.s001.pptx]

## Slide 1
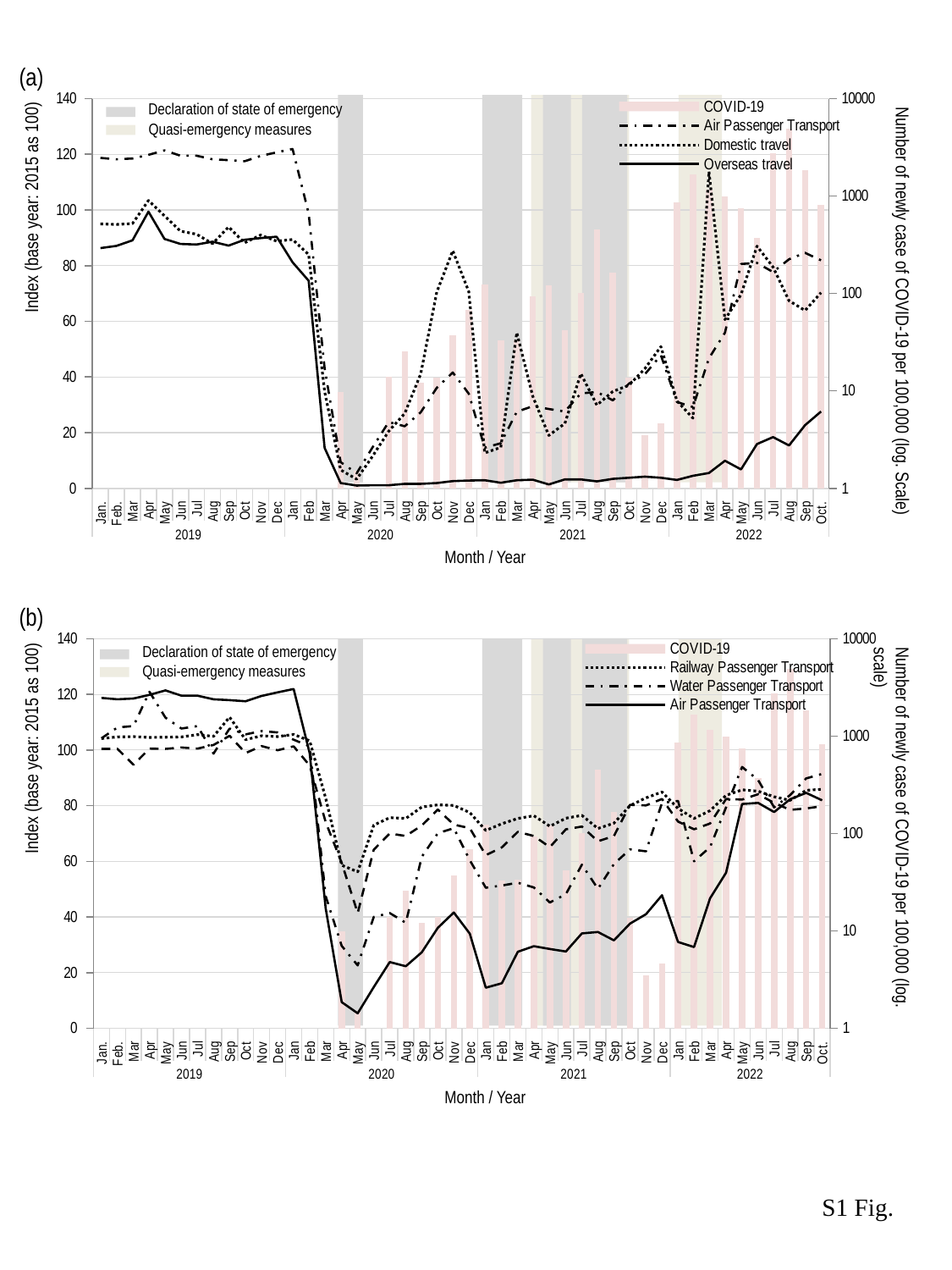

(a)
### Chart
| Category | COVID-19 | Air Passenger Transport | Domestic travel | Overseas travel |
|---|---|---|---|---|
| Jan. | 0.0 | 118.7 | 95.0 | 86.3 |
| Feb. | 0.0 | 118.2 | 94.8 | 87.1 |
| Mar | 0.0 | 118.5 | 95.1 | 89.1 |
| Apr | 0.0 | 119.8 | 103.4 | 99.4 |
| May | 0.0 | 121.4 | 97.9 | 89.6 |
| Jun | 0.0 | 119.5 | 92.4 | 87.8 |
| Jul | 0.0 | 119.5 | 91.3 | 87.6 |
| Aug | 0.0 | 118.2 | 87.9 | 88.6 |
| Sep | 0.0 | 117.9 | 93.9 | 87.2 |
| Oct | 0.0 | 117.5 | 88.2 | 89.3 |
| Nov | 0.0 | 119.4 | 91.0 | 89.9 |
| Dec | 0.0 | 120.7 | 88.8 | 90.4 |
| Jan | 0.0 | 121.9 | 89.4 | 81.2 |
| Feb | 0.0 | 98.8 | 84.0 | 74.6 |
| Mar | 1.0 | 43.1 | 34.8 | 14.5 |
| Apr | 9.799999999999999 | 9.4 | 6.6 | 1.9 |
| May | 1.5000000000000004 | 5.4 | 3.3 | 1.0 |
| Jun | 0.8999999999999999 | 14.8 | 11.6 | 1.1 |
| Jul | 13.899999999999999 | 23.8 | 20.5 | 1.1 |
| Aug | 25.599999999999994 | 22.3 | 26.9 | 1.6 |
| Sep | 12.100000000000001 | 27.3 | 41.1 | 1.6 |
| Oct | 13.799999999999999 | 36.1 | 70.5 | 1.9 |
| Nov | 37.300000000000004 | 41.6 | 85.4 | 2.6 |
| Dec | 68.49999999999999 | 34.0 | 70.7 | 2.8 |
| Jan | 122.79999999999997 | 14.6 | 12.7 | 2.9 |
| Feb | 33.0 | 16.2 | 14.7 | 2.0 |
| Mar | 33.2 | 27.5 | 55.9 | 2.9 |
| Apr | 93.0 | 29.5 | 33.0 | 3.1 |
| May | 121.59999999999998 | 28.5 | 19.0 | 1.4 |
| Jun | 41.79999999999999 | 27.6 | 23.3 | 3.2 |
| Jul | 100.49999999999997 | 34.1 | 41.2 | 3.2 |
| Aug | 449.9 | 34.6 | 29.9 | 2.5 |
| Sep | 164.89999999999998 | 31.6 | 34.8 | 3.4 |
| Oct | 13.6 | 37.6 | 37.0 | 3.8 |
| Nov | 3.5000000000000013 | 41.0 | 43.0 | 4.2 |
| Dec | 4.600000000000001 | 47.8 | 50.9 | 3.8 |
| Jan | 858.3000000000001 | 31.0 | 31.4 | 3.0 |
| Feb | 1669.5 | 29.2 | 25.2 | 4.5 |
| Mar | 1169.5000000000005 | 46.7 | 113.8 | 5.5 |
| Apr | 990.9 | 55.9 | 60.6 | 9.9 |
| May | 745.6999999999999 | 80.6 | 69.6 | 6.8 |
| Jun | 371.8 | 81.0 | 87.0 | 15.9 |
| Jul | 2745.3 | 77.7 | 79.5 | 18.4 |
| Aug | 4893.700000000002 | 82.3 | 67.4 | 15.4 |
| Sep | 1841.1999999999994 | 84.6 | 63.9 | 22.7 |
| Oct. | 817.4999999999999 | 81.9 | 70.3 | 27.6 |Declaration of state of emergency
Quasi-emergency measures
Index (base year: 2015 as 100)
Number of newly case of COVID-19 per 100,000 (log. Scale)
Month / Year
(b)
### Chart
| Category | COVID-19 | Railway Passenger Transport | Water Passenger Transport | Air Passenger Transport | Road Passenger Transport |
|---|---|---|---|---|---|
| Jan. | 0.0 | 104.1 | 104.2 | 118.7 | 100.4 |
| Feb. | 0.0 | 104.7 | 108.1 | 118.2 | 100.4 |
| Mar | 0.0 | 104.8 | 108.6 | 118.5 | 94.7 |
| Apr | 0.0 | 104.5 | 120.9 | 119.8 | 100.5 |
| May | 0.0 | 104.6 | 111.6 | 121.4 | 100.4 |
| Jun | 0.0 | 104.7 | 107.7 | 119.5 | 100.9 |
| Jul | 0.0 | 105.5 | 108.6 | 119.5 | 100.5 |
| Aug | 0.0 | 104.9 | 98.7 | 118.2 | 101.8 |
| Sep | 0.0 | 111.8 | 107.7 | 117.9 | 105.1 |
| Oct | 0.0 | 103.7 | 105.6 | 117.5 | 98.9 |
| Nov | 0.0 | 105.1 | 106.8 | 119.4 | 101.4 |
| Dec | 0.0 | 104.8 | 106.3 | 120.7 | 99.9 |
| Jan | 0.0 | 105.6 | 103.7 | 121.9 | 101.3 |
| Feb | 0.0 | 103.3 | 101.3 | 98.8 | 94.4 |
| Mar | 1.0 | 82.7 | 47.5 | 43.1 | 74.1 |
| Apr | 9.799999999999999 | 58.6 | 29.6 | 9.4 | 59.4 |
| May | 1.5000000000000004 | 56.2 | 22.6 | 5.4 | 41.4 |
| Jun | 0.8999999999999999 | 73.0 | 40.0 | 14.8 | 64.2 |
| Jul | 13.899999999999999 | 75.7 | 41.4 | 23.8 | 70.0 |
| Aug | 25.599999999999994 | 75.4 | 37.9 | 22.3 | 69.1 |
| Sep | 12.100000000000001 | 79.5 | 61.6 | 27.3 | 72.9 |
| Oct | 13.799999999999999 | 80.3 | 70.1 | 36.1 | 78.6 |
| Nov | 37.300000000000004 | 80.1 | 71.9 | 41.6 | 73.2 |
| Dec | 68.49999999999999 | 77.5 | 60.4 | 34.0 | 71.9 |
| Jan | 122.79999999999997 | 71.1 | 50.5 | 14.6 | 62.2 |
| Feb | 33.0 | 73.5 | 51.3 | 16.2 | 65.0 |
| Mar | 33.2 | 75.4 | 52.3 | 27.5 | 70.6 |
| Apr | 93.0 | 76.4 | 50.6 | 29.5 | 69.1 |
| May | 121.59999999999998 | 72.6 | 45.2 | 28.5 | 65.1 |
| Jun | 41.79999999999999 | 75.5 | 48.2 | 27.6 | 71.5 |
| Jul | 100.49999999999997 | 76.5 | 58.8 | 34.1 | 72.5 |
| Aug | 449.9 | 71.8 | 50.1 | 34.6 | 67.2 |
| Sep | 164.89999999999998 | 73.7 | 59.0 | 31.6 | 69.1 |
| Oct | 13.6 | 80.0 | 64.3 | 37.6 | 80.3 |
| Nov | 3.5000000000000013 | 82.8 | 63.6 | 41.0 | 80.1 |
| Dec | 4.600000000000001 | 84.9 | 81.0 | 47.8 | 82.4 |
| Jan | 858.3000000000001 | 79.0 | 81.7 | 31.0 | 74.3 |
| Feb | 1669.5 | 75.4 | 60.0 | 29.2 | 71.5 |
| Mar | 1169.5000000000005 | 78.2 | 65.1 | 46.7 | 73.6 |
| Apr | 990.9 | 83.7 | 79.4 | 55.9 | 82.4 |
| May | 745.6999999999999 | 85.7 | 93.9 | 80.6 | 82.2 |
| Jun | 371.8 | 85.2 | 89.2 | 81.0 | 84.1 |
| Jul | 2745.3 | 83.2 | 79.4 | 77.7 | 81.0 |
| Aug | 4893.700000000002 | 81.9 | 83.8 | 82.3 | 78.5 |
| Sep | 1841.1999999999994 | 85.5 | 89.8 | 84.6 | 79.0 |
| Oct. | 817.4999999999999 | 85.9 | 91.4 | 81.9 | 79.8 |Declaration of state of emergency
Quasi-emergency measures
Index (base year: 2015 as 100)
Number of newly case of COVID-19 per 100,000 (log. scale)
Month / Year
S1 Fig.
