# Supplementary material for: Effect of voluntary human mobility restrictions on vector-borne diseases during the COVID-19 pandemic in Japan: A descriptive epidemiological study using a national database (2016 to 2021)
Source: PLoS One. 2023 May 25;18(5):e0285107. doi: 10.1371/journal.pone.0285107 (PMC10212128; doi:10.1371/journal.pone.0285107)
Supplement: S2 Fig — Figures indicate the number of outpatients who visited the clinic by month. The declaration of state of emergency was in effect from April 7 to May 25, 2020; from January 8 to March 21, 2021; from April 25 to June 20, 2021; and from July 12 to September 30, 2021. In addition, quasi-emergency measures were in effect from April 5 to September 30, 2021. (PPTX) [file pone.0285107.s002.pptx]

## Slide 1
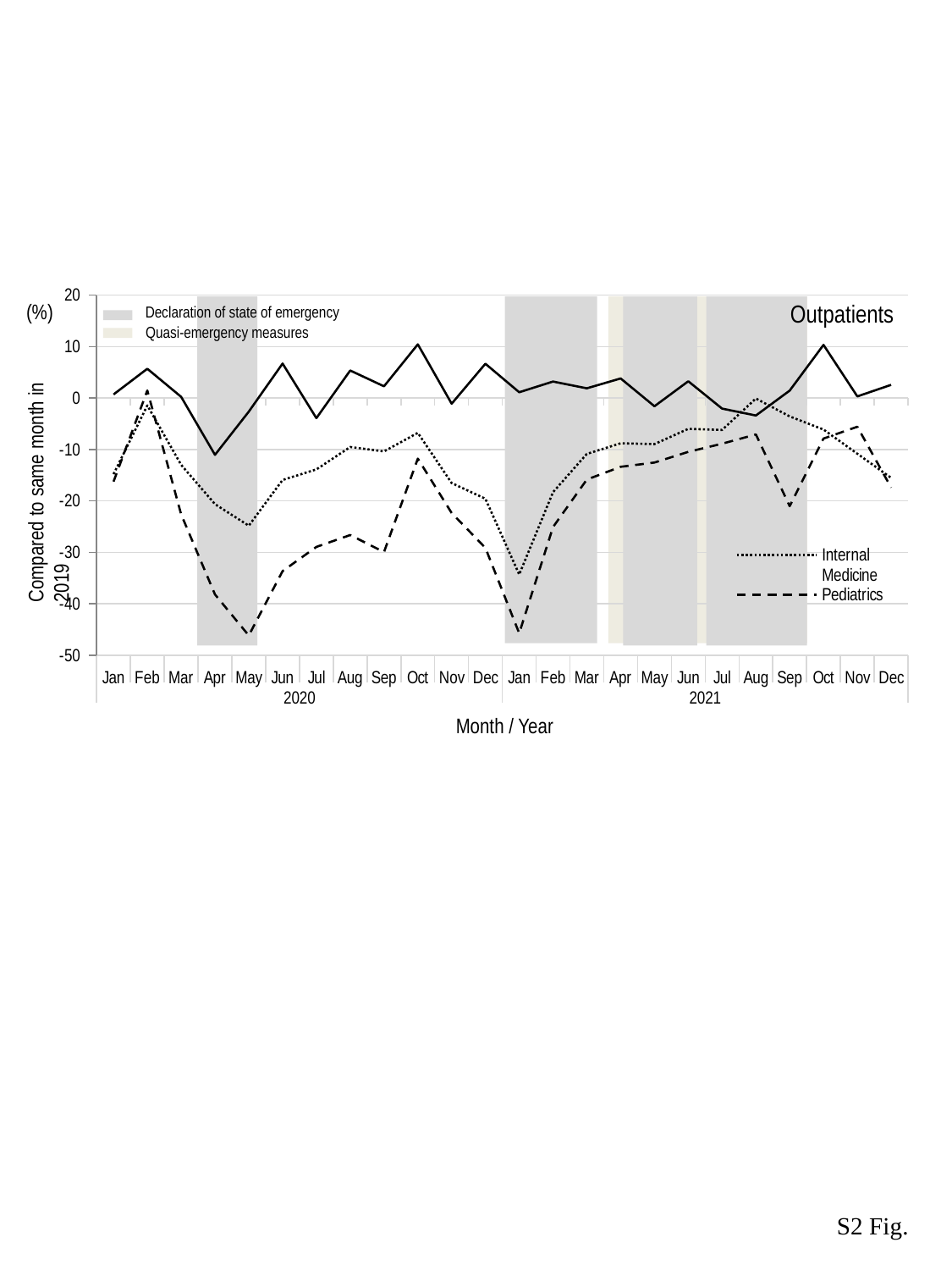

### Chart
| Category | Internal Medicine | Pediatrics | Dermatlogy |
|---|---|---|---|
| Jan | -14.785577638774484 | -16.231320567033393 | 0.7041644510111669 |
| Feb | -1.4304290238624204 | 1.4718004280435313 | 5.699510242071011 |
| Mar | -12.974542021267949 | -22.61697828537245 | 0.24173524829080276 |
| Apr | -20.623490814636607 | -38.17156439527909 | -11.03270492657015 |
| May | -24.809209366272654 | -46.150946534197914 | -2.6380346712215874 |
| Jun | -15.912579990423964 | -33.63192051414579 | 6.707918560875442 |
| Jul | -13.857548588101306 | -28.930956434638837 | -3.8982853923627205 |
| Aug | -9.509130805210306 | -26.622062733911804 | 5.361203533197104 |
| Sep | -10.358211473148739 | -29.928587763359076 | 2.286280446366026 |
| Oct | -6.753212362764745 | -11.773043196885054 | 10.442883116688034 |
| Nov | -16.506740001113194 | -22.33628267857044 | -1.0942120030117892 |
| Dec | -19.56979580598334 | -29.11780786195718 | 6.671243161472439 |
| Jan | -34.29211208756949 | -45.712770338975304 | 1.133648953075943 |
| Feb | -18.284728791260335 | -25.084566652683556 | 3.2105983301148777 |
| Mar | -10.842894113844503 | -15.834327941124807 | 1.904652560879495 |
| Apr | -8.789928586050353 | -13.35526580872186 | 3.8197206851236682 |
| May | -8.945882478484881 | -12.531462916161287 | -1.5906388180693547 |
| Jun | -5.976505991208012 | -10.44388367794129 | 3.271772984520581 |
| Jul | -6.190602079102638 | -8.860040426912924 | -2.0459635794716635 |
| Aug | -0.08443548403806746 | -7.065932328407157 | -3.3874043976506893 |
| Sep | -3.562213579130022 | -21.00620157961207 | 1.4575846070965353 |
| Oct | -6.114383501640701 | -7.859303255343786 | 10.335653370601833 |
| Nov | -10.81948170863027 | -5.566051838865242 | 0.3245200066640488 |
| Dec | -15.499411093339782 | -17.386182975488985 | 2.5759700467948923 |Outpatients
(%)
Declaration of state of emergency
Quasi-emergency measures
Compared to same month in 2019
Month / Year
S2 Fig.
