# Supplementary material for: Effect of voluntary human mobility restrictions on vector-borne diseases during the COVID-19 pandemic in Japan: A descriptive epidemiological study using a national database (2016 to 2021)
Source: PLoS One. 2023 May 25;18(5):e0285107. doi: 10.1371/journal.pone.0285107 (PMC10212128; doi:10.1371/journal.pone.0285107)
Supplement: S3 Fig — (a) Changes in the number of foreign nationals entering Japan between January 2016 and December 2021. The filled rectangle represents the period of the Olympic Games in Tokyo (from July 23 to August 8, 2021). The unfilled rectangle represents the period of the Paralympic Games in Tokyo (from August 24 to September 5, 2021). (b) Changes in the number of Japanese returnees to Japan from January 2016 to December 2021. (c) Changes in the actual amount of loaded container cargo volume handled within foreign trade from 2016 to 2021. (PPTX) [file pone.0285107.s003.pptx]

## Slide 1
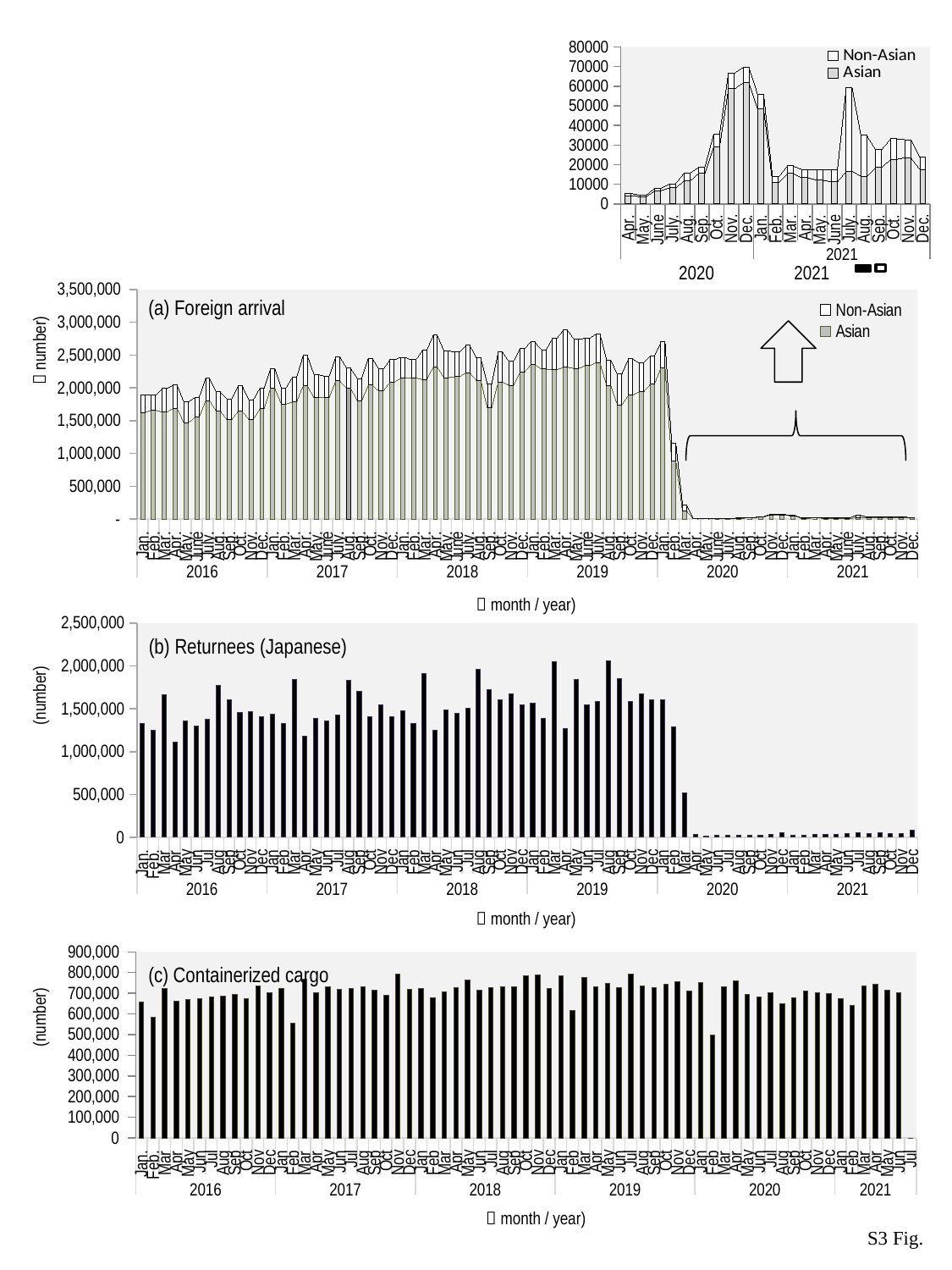

### Chart
| Category | Asian | Non-Asian |
|---|---|---|
| Apr. | 4034.0 | 1278.0 |
| May. | 3766.0 | 722.0 |
| June | 6754.0 | 1274.0 |
| July. | 8241.0 | 2059.0 |
| Aug. | 11868.0 | 4014.0 |
| Sep. | 15724.0 | 3137.0 |
| Oct. | 29220.0 | 6358.0 |
| Nov. | 58669.0 | 7934.0 |
| Dec. | 61868.0 | 7874.0 |
| Jan. | 48317.0 | 7401.0 |
| Feb. | 10870.0 | 2962.0 |
| Mar. | 15565.0 | 3833.0 |
| Apr. | 13303.0 | 4255.0 |
| May. | 12163.0 | 5213.0 |
| June | 11245.0 | 6040.0 |
| July. | 16777.0 | 42688.0 |
| Aug. | 14123.0 | 20842.0 |
| Sep. | 18634.0 | 9122.0 |
| Oct. | 22712.0 | 10516.0 |
| Nov. | 23611.0 | 9140.0 |
| Dec. | 17612.0 | 6173.0 |(a’)
2020 2021
### Chart
| Category | Asian | Non-Asian |
|---|---|---|
| Jan. | 1627068.0 | 262072.0 |
| Feb. | 1661398.0 | 229005.0 |
| Mar. | 1629434.0 | 362986.0 |
| Apr. | 1690796.0 | 353061.0 |
| May. | 1468474.0 | 317902.0 |
| June | 1550057.0 | 304091.0 |
| July. | 1805247.0 | 339513.0 |
| Aug. | 1647948.0 | 292054.0 |
| Sep. | 1512841.0 | 316497.0 |
| Oct. | 1650321.0 | 383051.0 |
| Nov. | 1519950.0 | 296658.0 |
| Dec. | 1687866.0 | 310622.0 |
| Jan. | 1997437.0 | 297480.0 |
| Feb. | 1752810.0 | 245154.0 |
| Mar. | 1788372.0 | 375997.0 |
| Apr. | 2035461.0 | 459901.0 |
| May. | 1853698.0 | 353282.0 |
| June | 1850052.0 | 325834.0 |
| July. | 2110277.0 | 368126.0 |
| Aug. | 1999173.0 | 305824.0 |
| Sep. | 1804870.0 | 336848.0 |
| Oct. | 2042481.0 | 407362.0 |
| Nov. | 1953006.0 | 336076.0 |
| Dec. | 2089910.0 | 339351.0 |
| Jan. | 2147314.0 | 318838.0 |
| Feb. | 2154067.0 | 274415.0 |
| Mar. | 2129852.0 | 447887.0 |
| Apr. | 2321195.0 | 482583.0 |
| May. | 2155640.0 | 406688.0 |
| June | 2171531.0 | 378620.0 |
| July. | 2231854.0 | 416047.0 |
| Aug. | 2112838.0 | 343546.0 |
| Sep. | 1696376.0 | 359852.0 |
| Oct. | 2091086.0 | 458220.0 |
| Nov. | 2038581.0 | 366353.0 |
| Dec. | 2234370.0 | 364349.0 |
| Jan. | 2358452.0 | 344108.0 |
| Feb. | 2288283.0 | 293487.0 |
| Mar. | 2274609.0 | 477585.0 |
| Apr. | 2316171.0 | 569285.0 |
| May. | 2295773.0 | 447046.0 |
| June | 2340400.0 | 413583.0 |
| July. | 2382612.0 | 442165.0 |
| Aug. | 2039889.0 | 385941.0 |
| Sep. | 1742625.0 | 466375.0 |
| Oct. | 1892754.0 | 548858.0 |
| Nov. | 1938621.0 | 446116.0 |
| Dec. | 2055377.0 | 427064.0 |
| Jan. | 2308263.0 | 390561.0 |
| Feb. | 890951.0 | 265009.0 |
| Mar. | 124682.0 | 92997.0 |
| Apr. | 4034.0 | 1278.0 |
| May. | 3766.0 | 722.0 |
| June | 6754.0 | 1274.0 |
| July. | 8241.0 | 2059.0 |
| Aug. | 11868.0 | 4014.0 |
| Sep. | 15724.0 | 3137.0 |
| Oct. | 29220.0 | 6358.0 |
| Nov. | 58669.0 | 7934.0 |
| Dec. | 61868.0 | 7874.0 |
| Jan. | 48317.0 | 7401.0 |
| Feb. | 10870.0 | 2962.0 |
| Mar. | 15565.0 | 3833.0 |
| Apr. | 13303.0 | 4255.0 |
| May. | 12163.0 | 5213.0 |
| June | 11245.0 | 6040.0 |
| July. | 16777.0 | 42688.0 |
| Aug. | 14123.0 | 20842.0 |
| Sep. | 18634.0 | 9122.0 |
| Oct. | 22712.0 | 10516.0 |
| Nov. | 23611.0 | 9140.0 |
| Dec. | 17612.0 | 6173.0 |(a) Foreign arrival
（number)
（month / year)
### Chart
| Category | No. of returnees |
|---|---|
| Jan. | 1327934.0 |
| Feb. | 1249714.0 |
| Mar | 1666757.0 |
| Apr | 1112783.0 |
| May | 1355176.0 |
| Jun | 1294226.0 |
| Jul | 1374293.0 |
| Aug | 1774634.0 |
| Sep | 1604107.0 |
| Oct | 1456787.0 |
| Nov | 1468345.0 |
| Dec | 1403496.0 |
| Jan | 1436386.0 |
| Feb | 1331819.0 |
| Mar | 1843939.0 |
| Apr | 1183666.0 |
| May | 1388836.0 |
| Jun | 1360735.0 |
| Jul | 1422671.0 |
| Aug | 1833092.0 |
| Sep | 1704807.0 |
| Oct | 1410813.0 |
| Nov | 1549609.0 |
| Dec | 1410080.0 |
| Jan | 1479500.0 |
| Feb | 1325270.0 |
| Mar | 1912124.0 |
| Apr | 1252052.0 |
| May | 1481754.0 |
| Jun | 1448600.0 |
| Jul | 1504890.0 |
| Aug | 1964601.0 |
| Sep | 1719802.0 |
| Oct | 1601792.0 |
| Nov | 1675859.0 |
| Dec | 1542710.0 |
| Jan | 1567008.0 |
| Feb | 1389824.0 |
| Mar | 2054155.0 |
| Apr | 1269871.0 |
| May | 1838301.0 |
| Jun | 1547666.0 |
| Jul | 1589288.0 |
| Aug | 2061962.0 |
| Sep | 1850064.0 |
| Oct | 1585490.0 |
| Nov | 1674511.0 |
| Dec | 1601915.0 |
| Jan | 1608306.0 |
| Feb | 1289648.0 |
| Mar | 521730.0 |
| Apr | 38983.0 |
| May | 14864.0 |
| Jun | 20615.0 |
| Jul | 27135.0 |
| Aug | 23939.0 |
| Sep | 23351.0 |
| Oct | 26645.0 |
| Nov | 30453.0 |
| Dec | 57601.0 |
| Jan | 25232.0 |
| Feb | 20994.0 |
| Mar | 38929.0 |
| Apr | 29795.0 |
| May | 32414.0 |
| Jun | 43441.0 |
| Jul | 51628.0 |
| Aug | 45555.0 |
| Sep | 52366.0 |
| Oct | 42209.0 |
| Nov | 48098.0 |
| Dec | 84536.0 |(b) Returnees (Japanese)
(number)
（month / year)
### Chart
| Category | No. of containers |
|---|---|
| Jan. | 656393.0 |
| Feb. | 583602.0 |
| Mar | 720997.0 |
| Apr | 662222.0 |
| May | 671700.0 |
| Jun | 672816.0 |
| Jul | 680971.0 |
| Aug | 685061.0 |
| Sep | 692514.0 |
| Oct | 673536.0 |
| Nov | 735747.0 |
| Dec | 703251.0 |
| Jan | 723968.0 |
| Feb | 556497.0 |
| Mar | 769802.0 |
| Apr | 701746.0 |
| May | 732330.0 |
| Jun | 717636.0 |
| Jul | 723790.0 |
| Aug | 731618.0 |
| Sep | 716258.0 |
| Oct | 691489.0 |
| Nov | 791748.0 |
| Dec | 720797.0 |
| Jan | 723452.0 |
| Feb | 677837.0 |
| Mar | 707444.0 |
| Apr | 727018.0 |
| May | 764479.0 |
| Jun | 714087.0 |
| Jul | 726086.0 |
| Aug | 729639.0 |
| Sep | 729476.0 |
| Oct | 783373.0 |
| Nov | 787878.0 |
| Dec | 721277.0 |
| Jan | 782965.0 |
| Feb | 615262.0 |
| Mar | 774579.0 |
| Apr | 731411.0 |
| May | 747670.0 |
| Jun | 728636.0 |
| Jul | 792012.0 |
| Aug | 734938.0 |
| Sep | 727095.0 |
| Oct | 742344.0 |
| Nov | 756294.0 |
| Dec | 712462.0 |
| Jan | 749824.0 |
| Feb | 497134.0 |
| Mar | 729271.0 |
| Apr | 760910.0 |
| May | 692489.0 |
| Jun | 680289.0 |
| Jul | 703889.0 |
| Aug | 648728.0 |
| Sep | 676734.0 |
| Oct | 710753.0 |
| Nov | 704642.0 |
| Dec | 698166.0 |
| Jan | 675034.0 |
| Feb | 641148.0 |
| Mar | 734125.0 |
| Apr | 744745.0 |
| May | 714479.0 |
| Jun | 703710.0 |
| Jul | 0.0 |(c) Containerized cargo
(number)
（month / year)
S3 Fig.
