# Supplementary material for: Effect of voluntary human mobility restrictions on vector-borne diseases during the COVID-19 pandemic in Japan: A descriptive epidemiological study using a national database (2016 to 2021)
Source: PLoS One. 2023 May 25;18(5):e0285107. doi: 10.1371/journal.pone.0285107 (PMC10212128; doi:10.1371/journal.pone.0285107)
Supplement: S4 Fig — SFTS: severe fever with thrombocytopenia syndrome. (PPTX) [file pone.0285107.s004.pptx]

## Slide 1
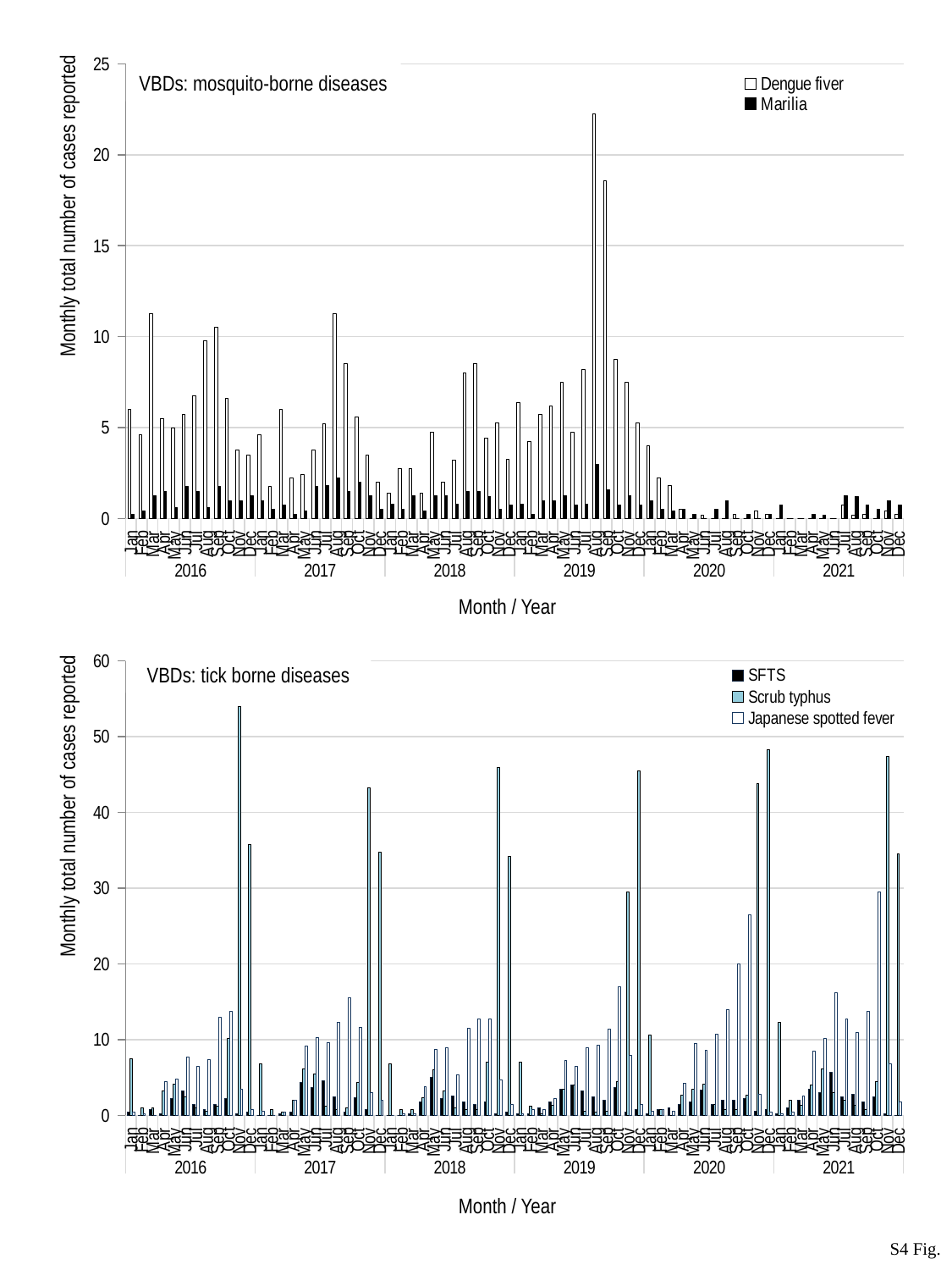

### Chart
| Category | Dengue fiver | Marilia |
|---|---|---|
| Jan | 6.0 | 0.25 |
| Feb | 4.6 | 0.4 |
| Mar | 11.25 | 1.25 |
| Apr | 5.5 | 1.5 |
| May | 5.0 | 0.6 |
| Jun | 5.75 | 1.75 |
| Jul | 6.75 | 1.5 |
| Aug | 9.8 | 0.6 |
| Sep | 10.5 | 1.75 |
| Oct | 6.6 | 1.0 |
| Nov | 3.75 | 1.0 |
| Dec | 3.5 | 1.25 |
| Jan | 4.6 | 1.0 |
| Feb | 1.75 | 0.5 |
| Mar | 6.0 | 0.75 |
| Apr | 2.25 | 0.25 |
| May | 2.4 | 0.4 |
| Jun | 3.75 | 1.75 |
| Jul | 5.2 | 1.8 |
| Aug | 11.25 | 2.25 |
| Sep | 8.5 | 1.5 |
| Oct | 5.6 | 2.0 |
| Nov | 3.5 | 1.25 |
| Dec | 2.0 | 0.5 |
| Jan | 1.4 | 0.8 |
| Feb | 2.75 | 0.5 |
| Mar | 2.75 | 1.25 |
| Apr | 1.4 | 0.4 |
| May | 4.75 | 1.25 |
| Jun | 2.0 | 1.25 |
| Jul | 3.2 | 0.8 |
| Aug | 8.0 | 1.5 |
| Sep | 8.5 | 1.5 |
| Oct | 4.4 | 1.2 |
| Nov | 5.25 | 0.5 |
| Dec | 3.25 | 0.75 |
| Jan | 6.4 | 0.8 |
| Feb | 4.25 | 0.25 |
| Mar | 5.75 | 1.0 |
| Apr | 6.2 | 1.0 |
| May | 7.5 | 1.25 |
| Jun | 4.75 | 0.75 |
| Jul | 8.2 | 0.8 |
| Aug | 22.25 | 3.0 |
| Sep | 18.6 | 1.6 |
| Oct | 8.75 | 0.75 |
| Nov | 7.5 | 1.25 |
| Dec | 5.25 | 0.75 |
| Jan | 4.0 | 1.0 |
| Feb | 2.25 | 0.5 |
| Mar | 1.8 | 0.4 |
| Apr | 0.5 | 0.5 |
| May | 0.0 | 0.25 |
| Jun | 0.2 | 0.0 |
| Jul | 0.0 | 0.5 |
| Aug | 0.0 | 1.0 |
| Sep | 0.25 | 0.0 |
| Oct | 0.0 | 0.25 |
| Nov | 0.4 | 0.0 |
| Dec | 0.25 | 0.25 |
| Jan | 0.0 | 0.75 |
| Feb | 0.0 | 0.0 |
| Mar | 0.0 | 0.0 |
| Apr | 0.0 | 0.25 |
| May | 0.0 | 0.2 |
| Jun | 0.0 | 0.0 |
| Jul | 0.75 | 1.25 |
| Aug | 0.2 | 1.2 |
| Sep | 0.25 | 0.75 |
| Oct | 0.0 | 0.5 |
| Nov | 0.4 | 1.0 |
| Dec | 0.25 | 0.75 |VBDs: mosquito-borne diseases
 Monthly total number of cases reported
Month / Year
### Chart
| Category | SFTS | Scrub typhus | Japanese spotted fever |
|---|---|---|---|
| Jan | 0.5 | 7.5 | 0.5 |
| Feb | 0.0 | 1.0 | 0.2 |
| Mar | 0.75 | 1.0 | 0.0 |
| Apr | 0.25 | 3.25 | 4.5 |
| May | 2.2 | 4.2 | 4.8 |
| Jun | 3.25 | 2.5 | 7.75 |
| Jul | 1.5 | 1.0 | 6.5 |
| Aug | 0.8 | 0.6 | 7.4 |
| Sep | 1.5 | 1.25 | 13.0 |
| Oct | 2.2 | 10.2 | 13.8 |
| Nov | 0.25 | 54.0 | 3.5 |
| Dec | 0.5 | 35.75 | 0.75 |
| Jan | 0.0 | 6.8 | 0.6 |
| Feb | 0.0 | 0.75 | 0.0 |
| Mar | 0.25 | 0.5 | 0.5 |
| Apr | 0.5 | 2.0 | 2.0 |
| May | 4.4 | 6.2 | 9.2 |
| Jun | 3.75 | 5.5 | 10.25 |
| Jul | 4.6 | 1.2 | 9.6 |
| Aug | 2.5 | 0.75 | 12.25 |
| Sep | 0.5 | 1.0 | 15.5 |
| Oct | 2.4 | 4.4 | 11.6 |
| Nov | 0.75 | 43.25 | 3.0 |
| Dec | 0.0 | 34.75 | 2.0 |
| Jan | 0.0 | 6.8 | 0.0 |
| Feb | 0.0 | 0.75 | 0.25 |
| Mar | 0.25 | 0.75 | 0.25 |
| Apr | 1.8 | 2.4 | 3.8 |
| May | 5.0 | 6.0 | 8.75 |
| Jun | 2.25 | 3.25 | 9.0 |
| Jul | 2.6 | 1.0 | 5.4 |
| Aug | 1.75 | 0.75 | 11.5 |
| Sep | 1.5 | 0.75 | 12.75 |
| Oct | 1.8 | 7.0 | 12.8 |
| Nov | 0.25 | 46.0 | 4.75 |
| Dec | 0.5 | 34.25 | 1.5 |
| Jan | 0.2 | 7.0 | 0.2 |
| Feb | 0.25 | 1.25 | 0.75 |
| Mar | 1.0 | 0.25 | 0.75 |
| Apr | 1.8 | 1.4 | 2.2 |
| May | 3.5 | 3.5 | 7.25 |
| Jun | 4.0 | 4.0 | 6.5 |
| Jul | 3.2 | 0.6 | 9.0 |
| Aug | 2.5 | 0.5 | 9.25 |
| Sep | 2.0 | 0.6 | 11.4 |
| Oct | 3.75 | 4.5 | 17.0 |
| Nov | 0.5 | 29.5 | 8.0 |
| Dec | 0.75 | 45.5 | 1.5 |
| Jan | 0.2 | 10.6 | 0.6 |
| Feb | 0.75 | 0.75 | 0.75 |
| Mar | 1.0 | 0.0 | 0.6 |
| Apr | 1.5 | 2.75 | 4.25 |
| May | 1.75 | 3.5 | 9.5 |
| Jun | 3.4 | 4.2 | 8.6 |
| Jul | 1.5 | 1.5 | 10.75 |
| Aug | 2.0 | 0.8 | 14.0 |
| Sep | 2.0 | 0.75 | 20.0 |
| Oct | 2.25 | 2.75 | 26.5 |
| Nov | 0.6 | 43.8 | 2.8 |
| Dec | 0.75 | 48.25 | 0.5 |
| Jan | 0.25 | 12.25 | 0.25 |
| Feb | 1.0 | 2.0 | 0.5 |
| Mar | 2.0 | 1.4 | 2.6 |
| Apr | 3.5 | 4.0 | 8.5 |
| May | 3.0 | 6.2 | 10.2 |
| Jun | 5.75 | 3.0 | 16.25 |
| Jul | 2.5 | 2.0 | 12.75 |
| Aug | 2.8 | 1.4 | 11.0 |
| Sep | 1.75 | 0.75 | 13.75 |
| Oct | 2.5 | 4.5 | 29.5 |
| Nov | 0.2 | 47.4 | 6.8 |
| Dec | 0.0 | 34.5 | 1.75 |VBDs: tick borne diseases
 Monthly total number of cases reported
Month / Year
S4 Fig.
