# Supplementary material for: Effect of voluntary human mobility restrictions on vector-borne diseases during the COVID-19 pandemic in Japan: A descriptive epidemiological study using a national database (2016 to 2021)
Source: PLoS One. 2023 May 25;18(5):e0285107. doi: 10.1371/journal.pone.0285107 (PMC10212128; doi:10.1371/journal.pone.0285107)
Supplement: Attachment — Submitted filename: Responce to Reviewers.docx [file pone.0285107.s016.docx]

Review Comments to the Author

Reviewer #1: The authors have made significant revisions to the manuscript to justify model assumptions and hypotheses. In particular, data on healthcare visits suggest that limited access may not be an important source of bias in the analysis and mobility data help to illustrate why 2020-2021 was defined as the pandemic period. The paper presents interesting analyses regarding how and why nonpharmaceutical interventions in Japan may have impacted VBD transmission. A few minor revisions, listed below, may help to provide further clarity.

Response to reviewers

Thank you for your valuable advices and comments. We have responded to each of your comments in detail.

C1. Given the amount of different analyses and dataset in this paper, additional structuring and explanation to signpost the different components may be helpful. For example, the final paragraph of the introduction could contain a brief list of the research questions or parts of the manuscript. As different data sources are introduced throughout the methods section, please provide a brief explanation of why they were collected (e.g., "the number of returnees to Japan…was examined to understand how travel impacts etc"). Additionally, clarify that restriction in access to healthcare was considered (line 222) to understand whether under ascertainment may have biased findings. The introduction should mention all analyses conducted, but currently does not clearly state the analysis that will be conducted on suspected region of infection.

R1a: We have listed the research questions and related topics in the final paragraph of the Introduction.

➡Line 83-97

The list of the research questions and the related topics in the present study is presented below:

1. Relationship between the incidences of VBDs and human mobility
A. Access to healthcare
B. Domestic passenger transportation activity
C. Foreign arrival
D. Returnees
E. Domestic human mobility: Long-distance travel / Population staying in local or national parks
2. Relationship between the incidences of VBDs and commodity distribution
A. Foreign trade
B. Domestic trade
3. Relationship between the incidences of VBDs and sex and age
4. Other relevant factors with the incidence of VBDs
A. COVID-19 epidemic status in Japan
B. Geographical factor: World region / domestic region

R1b. As you have indicated, we have described why we collected the figures in the respective paragraphs in the materials and methods section.

➡Line 130

Foreign arrivals were examined to understand how foreign travel impacts the incidence of VBDs.

➡Line 135

Returnees were examined to understand how foreign travel impacts the incidence of VBDs.

➡ Line 140-141

Cargo containers were examined to understand how overseas commodity distribution impacts the incidence of VBDs

➡Line 150-151

Domestic transport volumes were examined to determine the extent to which the long-distance movement of people and goods within a country affects the incidence of VBDs.

➡Line 177-178

Domestic human mobility was examined to understand the extent to which people's mobility in the country affected the incidence of VBDs.

➡Line 189-190

The frequency of access to healthcare was examined to understand whether underestimation may have biased findings.

R1b. We also mentioned on the suspected regions of infection in the introduction.

➡Line 62-68

It is known that the regional incidence of VBDs often depends on the habitat range of the vector. However, it is not clear whether these differences in the regional incidence are affected by changes in human mobility due to the pandemic of COVID-19. Since the incidence of malaria and dengue fever, in which humans are the source of infection, may correlate with human mobility, a reduction in the number of travelers from endemic areas may alter the suspected transmission area [7, 8]. In addition, several tick-borne diseases are rarely transmitted by humans, so the regional differences may not change.

C2: Although the revised title is helpful to indicate moderate behavior restriction, "voluntary" may be more clear than "non-coerceive."

R2: Thank you for your appropriate recommendation. We have revised "non-coercive" to "voluntary" in the title

➡Line 1-3

**Effect of voluntary human mobility restrictions on vector-borne diseases during the COVID-19 pandemic in Japan: A descriptive epidemiological study using a national database (2016 to 2021)**

C3: Line 68: The explanation provided in the discussion for potential sex differences is employment, but the hypothesis provided here involves "fear and anxiety." The suggested mechanism(s) should be consistent throughout.

R3: We have revised this paragraph assuming an association between sex and age and the incidence of VBDs to be consistent within this section of the discussion.

➡Line 75-81

It is unclear whether the sex- and age-specific incidence rates of VBDs changed during the COVID-19 pandemic. A study in Taiwan on scrub typhus considered the association between age groups and specific occupations, such as farmer or soldier [12]. Furthermore, the female population in China has a reportedly higher incidence of SFTS [13], which may be due to their greater exposure to ticks during agricultural work and susceptibility to the disease after infection [13]. As a high proportion of agricultural work is known to be the work at the time of infection with VBDs in Japan [14], the sex and age distributions of affected individuals may be influenced by the occupation.

**12.** Lee YS, Wang PH, Tseng SJ, Ko CF, Teng HJ. Epidemiology of scrub typhus in eastern Taiwan, 2000-2004. Jpn J Infect Dis. 2006;59:235-8. Available from: https://www.niid.go.jp/niid/ja/jjid.html

**13.** Qian J, Wei J, Ren L, Liu Y, Feng L. Sex differences in incidence and fatality of severe fever with thrombocytopenia syndrome: a comparative study based on national surveillance data of China. J Med Virol. 2023 Mar 3. doi: 10.1002/jmv.28632.

**14.** National Institute of Infectious Diseases. Scrub typhus and Japanese spotted fever in Japan 2007-2016. IASR. 2017;38: 109-112. Available from:

https://www.niid.go.jp/niid/en/basic-science/865-iasr/7342-448te.html

C4. Line 246: "our country" -> Japan

R4: We have revised “our country to Japan in the sentence.

➡Line 275-276

“The incidence of VBDs among notifiable infectious diseases during pre- and post-pandemic periods in Japan is shown in Table 1.”

C5. Line 248-50: The notation of giving the percentage with the smallest magnitude is a bit confusing. These values may be removed.

R5: We have removed the figures from the sentence as recommended.

➡Line 276-279

The incidence of Zika, Chikungunya fever, malaria, and dengue fevers, decreased by more than half during the pandemic compared to the pre-pandemic period. On the other hand, the incidence of relapsing fever, SFTS, scrub typhus, JSF, and Lyme disease increased during the pandemic compared to the pre-pandemic period.

C6. Table 1: There appear to be an errors in this table. For example, no cases of Eastern equine encephalitis were reported in either period but a 55% reduction is given, but dengue cases decreased and are listed as 0% change. Providing values for VBDs with no reported cases also appears extraneous and may make the table more difficult to read. These rows may be removed and a footnote may be used to list diseases with no reported cases.

R6: We apologize for the error which was a result of oversight. Appropriate revisions have been made and the columns that the case has not been reported has been changed to “n.r.” and it has been noted in the footnote that these diseases were not reported before or after the COVID-19 pandemic.

➡Line 280

C7. Line 285: "infected area" -> change to "suspected region of infection" as this is more clear

R7: Thank you for your appropriate suggestion. We have revised it accordingly

➡Line 314-315

We examined changes in the distribution of suspected regions of infection (world regions) for VBDs between the pre-pandemic and pandemic periods (Table 3).

C8. Line 285-94: The presentation and interpretation of the percentages here are unclear and potentially misleading. For example, line 290-1 says "the prevalence of dengue was also common in Asia (81.1%)," which I initially read to mean that 81.1% of people in the region were infected.

R8. The text has been revised for clarity.

➡Line 314-323

We examined changes in the distribution of suspected regions of infection (world regions) for VBDs between the pre-pandemic and pandemic periods (Table 3). Zika fever was reported in the pre-pandemic period, especially by travelers from Asia and the Americas, at a rate of over 40%; however, in the pandemic period, the number of cases decreased. Chikungunya fever was significantly more prevalent in visitors from Asia (91.7%) during the pre-pandemic period; however, the number of cases decreased during the pandemic period. Dengue fever was the most prevalent among visitors from Asia during the pre-pandemic period and the pandemic periods (87.3% and 81.1%, respectively). Malaria was especially common in visitors from Africa (75.1%) and Asia (14.2%) during the pre-pandemic period. Although these cases decreased during the pandemic period, the ratio was generally maintained (Africa 68.1% vs. Asia 12.8%).

C9. Line 336: "relevant factors" -> be more specific about the eliminated factors

R9. We have indicated the specific factors that we have excluded in the cited literature.

➡ Line 364-366

The decline in dengue cases in 2020 was associated with changes in human movement behaviors, excluding the climatic and immunological factors.

C10. Line 354: "may have had a significant impact on the incident" -> can this statement be supported using evidence from Table 3?

R10. This was an oversight error. This sentence has been appropriately corrected, as can be seen in Table 3.

➡Line 382-385

Although there was no significant decrease in the percentage of malarial infection among African visitors during the pandemic period, there was a significant decrease in the number of cases per year, from 42.25 (pre-pandemic) to 16.0 (pandemic). Thus, the decline in this number among African visitors may have a significant impact on the incidence of malaria in Japan.

C11. Line 358: "current Europe" -> unclear what this means, please rephrase

R11. The text has been revised to make it easier for readers to understand.

➡Line 389-391

It is not clear from this study whether the absence of infected travelers from Europe was due to low incidences of dengue fever, malaria and other VBDs in Europe or because the proportion of travelers was generally small.

C12. Line 373: "unlikely to have been affected" -> please provide further justification for this claim

R12. We have described the justification supporting this claim in the text.

➡Line 405-409

This claim may be supported by the fact that if tourists and others short-term visitors become infected with the tick-borne pathogen in the endemic area and, after an incubation period, develop the disease in the urban area, this will lead to an increase in the incidence in urban area. However, infection with VBDs occurred within the reporting prefecture for majority of the cases [14].

**14.** National Institute of Infectious Diseases. Scrub typhus and Japanese spotted fever in Japan 2007-2016. IASR. 2017;38: 109-112. Available from:

https://www.niid.go.jp/niid/en/basic-science/865-iasr/7342-448te.html

C13. Line 407-415: The information provided regarding fever and other clinical symptoms of the different VBDs appears to be addressing whether VBDs may be misdiagnosed as Covid, but the connection is unclear. Please provide additional interpretation of these results or remove them.

R13. The statement you have mentioned was added in the previous revision in response to a suggestion from an editor. However, we had deleted the sentence stating that it is difficult to differentiate symptoms of VBDs from those of COVID-19. What we really wanted to say here was that the rate of visits to medical facilities for emergency symptoms such as fever and rash was considered to be high. We have therefore added that in the revised text.

➡Line 442-449

However, the major mosquito-borne infectious diseases, dengue fever, Chikungunya fever, and Zika fever, if manifested, are characterized by fever and a generalized rash [53]. These infections are difficult to distinguish based on clinical symptoms. For tick-borne diseases, scrub typhus (n=4,185) and JSF (n=1,765), fever was observed in 95% and 99% of cases and rash in 86% and 94% of cases, respectively [54]. It is conceivable that the medical consultation rates for such emerging symptoms are not low. In addition, the majority of patients with tick-borne diseases were elderly individuals. Therefore, it is likely that the number of visits to the pediatric department was quite low, even during the non-pandemic period.

C14. Line 422: "age and sex were influenced by other factors" -> the direction of the hypothesized relationship is incorrect here.

R14. Thank you for pointing out this error. The entire paragraph has been revised, including the sentence you pointed out.

➡Line 456-459

Host factors such as age and sex differences were found to affect tick-borne diseases, but biological, social and behavioral factors must also be taken into account to determine the true impact. Mosquito-borne diseases, such as malaria, are reportedly modified by behavioral differences between men and women [55].

**55.** Okiring J, Epstein A, Namuganga JF, Kamya EV, Nabende I, Nassali M, et al. Gender difference in the incidenceof malaria diagnosed at public health facilities in Uganda. Malar J. 2022;21: 22. doi: 10.1186/s12936-022-04046-4

C15. Figure 4: The conceptual figure to indicate different covariates that may be relevant depending on the disease helps to organize the analysis, but the Venn Diagram format may be unnecessary to communicate this information. The figure could be improved by selecting a different format (perhaps a table), giving examples of specific covariates that fall under each covariate that were considered in the paper. If possible, it may also be helpful to include information about the hypothesized or estimated direction of the relationship.

R15. As this is a summary of the hypotheses found in this study, we would like for it to be presented as a diagram rather than a table. The use of doughnut charts instead of Venn diagrams eliminated the intersections of each confounding factor in the Venn diagram. However, it is not possible to say from this study about the proportion of segments in the doughnut charts, so it was assumed to be set equally.

➡Line 466

**Fig. 4. The hypothesized correlating factors influencing the incidence of vector-borne diseases**.

Reviewer #2:

Comment: The authors have addressed all the comments in previous review. My pleasure to recommend its acceptance.

Response to reviewer: We are delighted that you have recommended acceptance to PLoS ONE. We thank you very much for your time and critical comments which helped us significantly improve our manuscript.
